# The Role of the *MTUS1* Gene in the Development of Left Ventricular Noncompaction Cardiomyopathy—A Case Report

**DOI:** 10.3390/genes16020110

**Published:** 2025-01-21

**Authors:** Tevž Gorjanc, Jaka Šikonja, Ana Drole Torkar, Mojca Žerjav Tanšek, Jernej Kovač, Sara Bertok, Maruša Debeljak, Zvezdana Dolenc-Stražar, Marija Meznarič, Jernej Mlakar, Mirko Topalović, Gorazd Mlakar, Tadej Battelino, Urh Grošelj

**Affiliations:** 1 Faculty of Medicine, University of Ljubljana, SI-1000 Ljubljana, Slovenia; tevz.gorjanc@gmail.com (T.G.); jaka.sikonja@kclj.si (J.Š.); ana.droletorkar@kclj.si (A.D.T.); mojca.zerjav-tansek@mf.uni-lj.si (M.Ž.T.); jernej.kovac@kclj.si (J.K.); tadej.battelino@mf.uni-lj.si (T.B.); 2 Department of Endocrinology, Diabetes, and Metabolic Diseases, Division of Medicine, UMC Ljubljana, SI-1000 Ljubljana, Slovenia; 3 Department of Endocrinology, Diabetes, and Metabolic Diseases, University Children’s Hospital, UMC Ljubljana, SI-1000 Ljubljana, Slovenia; sara.bertok@kclj.si; 4 Center for Rare Diseases, University Children’s Hospital, UMC Ljubljana, SI-1000 Ljubljana, Slovenia; 5 Clinical Institute of Special Laboratory Diagnostics, University Children’s Hospital, UMC Ljubljana, SI-1000 Ljubljana, Slovenia; marusa.debeljak@kclj.si; 6 Institute of Pathology, Faculty of Medicine, University of Ljubljana, SI-1000 Ljubljana, Slovenia; zvezdana.dolenc@mf.uni-lj.si (Z.D.-S.); jernej.mlakar@kclj.si (J.M.); 7 Institute of Anatomy, Faculty of Medicine, University of Ljubljana, SI-1000 Ljubljana, Slovenia; marija.meznaric@mf.uni-lj.si; 8 Cardiology Department, University Children’s Hospital, UMC Ljubljana, SI-1000 Ljubljana, Slovenia; mirko.topalovic@kclj.si (M.T.); gorazd.mlakar@kclj.si (G.M.)

**Keywords:** microtubule-associated scaffold protein 1 (*MTUS1*), left ventricular noncompaction cardiomyopathy, whole-genome sequencing

## Abstract

Background/Objectives: The microtubule-associated scaffold protein 1 (*MTUS1*) gene affects the microtubule stability and cell polarity in the heart and could thus lead to the development of left ventricular noncompaction (LVNC). Pathological gene variants in *MTUS1* are associated with pathological phenotypes in both cell cultures and animal models. However, the literature lacks human studies on the specific effects of the *MTUS1* gene in heart disease, particularly in congenital LVNC. Methods: We present a case of a male infant, diagnosed with LVNC, who passed away at the age of 8 months due to end-stage heart failure. In the investigation process of the etiology of LVNC, whole-genome sequencing using next-generation sequencing was performed in the patient and his first-degree family members. Results: Genetic analysis identified two heterozygous variants in the *MTUS1* gene (NM_001363059.2:c.87C>G and NM_001363059.2:c.2449+421_2288-425del) in the presented patient. The first variant introduced an early stop codon, while the second caused the deletion of an entire exon, both of which significantly altered the protein structure. The older brother of the patient, at the age of 5 years, was a carrier of both variants; however, he was asymptomatic and without signs of heart disease on cardiac ultrasonography. Conclusions: Although, in theory, defects in the *MTUS1* gene may contribute to the development of LVNC, our observations indicate that *MTUS1* variants alone are not sufficient to cause LVNC or lead to any significant developmental disorder. Additional factors, whether genetic or environmental, are likely necessary for the clinical manifestation of LVNC.

## 1. Introduction

The microtubule-associated scaffold protein 1 (*MTUS1)* gene, located on chromosome 8p22, encodes multiple isoforms of angiotensin II receptor-interacting proteins (ATIP1/3/4) and plays a role in cardiovascular system development and function [1,2]. The protein ATIP3 influences microtubule stability, thereby regulating the structure and function of the cytoskeleton. Sustained microtubule stabilization negatively impacts cardiac function in cardiomyopathies by altering calcium handling and the distribution of cell organelles, while, conversely, microtubule destabilization is associated with maintained cardiac function in heart pathologies and improved heart failure outcomes in animal models [3,4]. Microtubule stability also serves a crucial role in influencing cell polarization, a fundamental process that is vital for myocardial compaction during cardiac embryogenesis [5]. Given its role in cytoskeleton regulation, *MTUS1* can impact cardiac embryogenesis and function throughout life [2,6].

Defects in the *MTUS1* gene have been associated with numerous diseases [7]. It exhibits antiproliferative effects and is considered a tumor suppressor gene; its reduced expression is associated with various types of malignancies, including breast, colorectal, pancreatic, lung, ovarian, and oral cancers [8,9]. Furthermore, its antiproliferative function is also significant in the cardiovascular system during both embryogenesis and in adulthood, as *MTUS1* knockout mice displayed a higher body mass throughout their lifespans, elevated blood pressure, spontaneous heart hypertrophy, and a shorter lifespan than wild-type mice. On the other hand, the overexpression of certain *MTUS1* gene transcripts reduced cardiac hypertrophic remodeling in mouse models subjected to pressure overload or phenylephrine treatment, preventing both a beneficial adaptive phase and a maladaptive phase of cardiac hypertrophy [2,7]. Additionally, the *MTUS1* gene decreases the microtubule stability in cell lines and may therefore have a role in advanced heart failure where microtubule overstabilization is seen [2,3,5]. The literature lacks human studies on the specific effects of the *MTUS1* gene in heart disease.

Left ventricular noncompaction cardiomyopathy (LVNC) is a distinct congenital cardiomyopathy characterized by abnormally prominent trabeculations and deep recesses within the wall of the LV [10]. The clinical features are heterogeneous, as patients may present with signs and symptoms of acute or chronic heart failure early in life or in adulthood; however, in the majority of cases, the condition remains asymptomatic [11]. While many genes have already been linked to LVNC [12], the role of *MTUS1* remains unclear. In a recent study, it was shown that, in cell lines, a genetic variant in the *MTUS1* gene reduces the extent of noncompaction by decreasing the microtubule stability and enhancing cell polarization; consequently, this effect could confer a protective role against LVNC, given the significance of cell polarity in the normal densification of the myocardium [5,6,11].

In this article, we aim to further explain the role of *MTUS1* in LVCN by presenting two brothers with identical heterozygous genetic variants in the *MTUS1* gene and a diametrical phenotype. While the first developed LVNC that led to lethal heart failure, the other remained asymptomatic without detectable heart disease.

## 2. Case Presentation

A male infant was referred to the University Children’s Hospital Ljubljana at seven months of age due to acute decompensation of dilated noncompaction cardiomyopathy. The patient was born to a Caucasian mother at 37 weeks of gestation. The pregnancy was uneventful, and he had a birth weight of 2440 g (3rd percentile), birth length of 45 cm (3rd percentile), and head circumference of 34.5 cm (25th percentile). His Apgar score was 9/9.

At the age of 3 months, he was diagnosed with bilateral cataracts that were surgically corrected. While suffering from a respiratory infection, a chest radiograph was performed at the age of 6 months, which revealed mild interstitial thickening together with cardiomegaly. In the following month, the boy became unusually irritable and was referred to the University Medical Centre Ljubljana due to the acute decompensation of heart failure with respiratory insufficiency. Initial laboratory tests revealed significantly elevated troponin (10.6 μg/I; reference range (RR): <0.1 μg/I), NT-pro-BNP (111011 ng/L; RR: <125.0 ng/L), and liver transaminases (ALT 11.51 µkat/L, RR: <0.58 µkat/L; AST 15.55 µkat/L, RR: <0.58 µkat/L). Parenteral diuretic therapy was immediately initiated; however, the condition progressed to cardiogenic shock.

Echocardiography revealed noncompaction dilated cardiomyopathy characterized by a dilated (LV diameter: 35 mm) and globally hypokinetic LV. The left ventricular ejection fraction, assessed using the M-Mode and BiPlane methods, was significantly reduced (27% and 14%, respectively). Hypertrabeculation of the apicolateral wall was observed on segmental analysis. The coexisting conditions included atrioventricular and ventriculoarterial concordance, mitral and tricuspid insufficiency, and an atrial septal defect with a patent foramen ovale. Cardiac MRI demonstrated marked left ventricular enlargement with severely compromised systolic function (ejection fraction < 10%). Pronounced trabeculations were observed in the lateral, inferior, and anterior walls. Diminished function and enlargement of the right ventricle were also observed. Despite aggressive intensive-care treatment with diuretics, multiple inotropic agents, and vasopressors, his condition continued to deteriorate, and he needed intubation and mechanical ventilation. Over the next few days, he was successfully resuscitated twice; however, in the following days, the patient passed away due to heart failure at the age of 8 months and 11 days.

The postmortem findings showed distinctive macroscopic and histological abnormalities consistent with noncompaction cardiomyopathy and focal dystrophic calcifications within the heart tissue. Heart examination also revealed characteristics of chronic hypoxic–ischemic injury. Furthermore, there were indications of fibrointimal hyperplasia within the coronary arteries, accompanied by the accumulation of a mucoid substance, which was also observed in the aortic valve.

**Figure 1:**
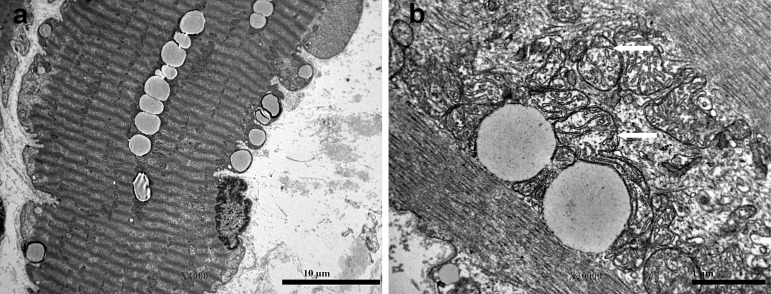
Electron microscopy of vastus lateralis muscle biopsy: (**a**) increased number of lipid droplets and subsarcolemmal glycogen in a hypercontracted muscle fiber; (**b**) arrows point to mitochondria with reduced number of cristae.

An ultrastructural analysis of the vastus lateralis muscle biopsy revealed normal sarcomeres along with lipid and glycogen accumulation, pointing to a metabolic disorder. Additionally, there were alterations in the mitochondrial structure, including variations in size, a reduced number of cristae, and the presence of giant mitochondria, which lacked paracrystalline inclusions (Figure 1). The enzyme activity of the respiratory chain complexes, determined at the Neurological Institute “Carlo Besta” I.R.C.C.S. Foundation in Milan, was normal.

Due to the deviations in the mitochondrial structure, suggestive of a mitochondrial disease, we performed next-generation sequencing (NGS) of the mitochondrial genome. We achieved 569-fold average horizontal coverage, allowing for the accurate detection of heteroplasmy levels as low as 10%, and it did not reveal any mitochondrial DNA pathological variants associated with the patient’s clinical phenotype.

Furthermore, we performed a whole-genome sequencing (WGS) trio analysis using NGS in the patient and both of his parents and analyzed genes included in the reference Human Genome 19 (HG19; also known as GRCh37), which revealed that the patient carried two heterozygous variants within the *MTUS1* gene. Both variants are classified as variants of unknown significance due to the lack of data. The first variant, NM_001363059.2:c.87C>G, was inherited from his mother and resulted in a significant molecular change, substituting tyrosine at position 29 with a termination codon (p.Tyr29Ter). Predictive algorithms (CADD) indicate a high likelihood of pathogenicity for this variant. The second variant, NM_001363059.2:c.2449+421_2288-425del, spans over the entire exon 4 within the *MTUS1* gene and results in a 1-kilobase deletion. This specific genetic change was inherited from his father. The clinical significance of this deletion remains unclear; however, it may be considered pathogenic due to a large-scale deletion. No relevant variants were identified in the analyzed genes in any of the three family members that could explain the clinical presentation, including those associated with cataract development, cardiomyopathies, and inborn errors of metabolism.

The patient’s father, aged 45, has an ascending aortic aneurysm that extends into the common carotid artery, which is currently being monitored. His echocardiography revealed dilation of the aortic bulbus and left ventricular wall hypertrophy. The mother, aged 39 years, has no known chronic comorbidities and her physical examination was normal.

The older brother, who is now 5 years old, showed no apparent signs of disease and was in good general health. Moreover, cardiac ultrasonography performed at the age of 3 did not reveal any cardiac anomalies. On the other hand, no eye disease or vision impairment was detected, including cataracts. This is particularly intriguing as he shared identical genetic variants in the *MTUS1* gene (c.87C>G and c.2449+421_2288-425del) with his younger brother. However, as siblings, they had a similar environmental background and shared approximately half of their genetic material.

## 3. Discussion

This study presents a case of an infant with LVNC and with heterozygous genetic variants in the *MTUS1* gene, who passed away at the age of 8 months due to end-stage heart failure.

Considered a congenital anomaly, LVNC has also been reported in acquired forms, occurring in response to physiological stressors such as exercise, dilated cardiomyopathy, and pregnancy [13,14]. In these cases, the presence of LVNC is likely a result of transient left ventricular dilatation, making milder preexisting forms of LVNC detectable on echocardiography [15]. During the first few weeks of uterine cardiac development, trabeculations and recesses in the ventricular wall increase the gas exchange area. By the eighth gestational week, these structures compress, and coronary circulation forms, resulting in a smooth ventricular wall. It is believed that any alterations in this compaction process can be causative for LVNC [16].

Among all patients who undergo cardiac ultrasonography, LVNC is observed in 0.014% [17]. Although LVNC can occur sporadically, approximately 30% of cases present as a familial condition among first-degree relatives, most commonly with autosomal dominant inheritance [18]. Several genes have been implicated in LVNC, including those responsible for encoding sarcomeric proteins (*TTN* and *MYH7*), cytoskeletal proteins (*P121L*), genes related to nuclear membrane components, chaperones, and the *TAFAZZIN* gene, causing Barth syndrome [16].

In pediatric cohorts, LVNC often presents with abnormal ECG, arrhythmias, chest pain, syncope, and sudden cardiac death. Pathological ECG findings were present in 87% of children with LVNC. A particularly poor prognosis was observed in cases where arrhythmia coexisted with morphological abnormalities and ventricular insufficiency [12].

The gene *MTUS1* encodes multiple ATIP protein isoforms that are widely expressed in normal human tissue. Owing to alternative splicing, three major ATIP isoforms have been identified in humans: ATIP1, ATIP3, and ATIP4 [1]. In normal and cancer cell lines, research has shown that ATIP3 and ATIP1 effectively suppress cell proliferation by inhibiting the growth factor-induced autophosphorylation of receptor tyrosine kinases and extracellular-regulated kinase and by lowering the reactive oxygen species levels in the mitochondria, which is an independent factor for cardiac hypertrophy [19,20,21].

The exact role of *MTUS1* in cardiac embryogenesis and later in life remains unclear in the current literature, and most of the studies were conducted on animals and cell models. The overexpression of *MTUS1* can lead to defects, primarily due to its influence on the cytoskeleton. Firstly, specific *MTUS1* isoform overexpression in adult mice causes left ventricular thinning, LV dysfunction, and reduced hypertrophic adaptation to artificially induced myocardial overload [2,7]. On the other hand, adult *MTUS1* knockout mice have elevated blood pressure and develop spontaneous heart hypertrophy compared to wild-type mice [7]. It is supposed that these observations are mainly due to *MTUS1*’s inhibitory effect on ERK signaling [2].

The pathogenesis of LVNC involves two key processes: (i) hypertrabeculation, characterized by excessive trabeculae formation, and (ii) noncompaction, the failure of trabeculae to remodel into a compact myocardial wall. Enhanced Notch signaling, through pathways involving *Neuregulin1*, *EphrinB2*, *BMP10*, *TBX20*, and *Sema3E*/*PlexinD1*, drives abnormal cardiomyocyte proliferation and these structural abnormalities [22,23]. Planar cell polarity (PCP), regulated by noncanonical Wnt signaling, plays a vital role in cardiomyocyte alignment and myocardial organization, with disruptions contributing to LVNC. Additionally, desmosomal proteins such as desmocollin 2 (DSC2) and desmoglein 2 (DSG2) are critical for cell–cell adhesion and polarization. Mutations in desmosomal genes and PCP pathways disturb cardiomyocyte polarity, mechanical coupling, and electrical signaling, further driving LVNC development [24].

Only one study has described the potential role of *MTUS1* in LVNC, where it was shown that the *MTUS1* genetic variant c.2617A>C (p.Asn873His) in HEK-293 cell lines could reduce the extent of noncompaction, since it decreases the microtubule stability and enhances the cell polarity through the decreased expression of α-tubulin and increased expression of α/β-tubulin heterodimer and PAR6 protein, and it also decreases the level of phosphorylated Rac1/Cdc42 [5]. In summary, *MTUS1* could influence LVNC through its impact on cell polarity and PCP signaling. Microtubules, which *MTUS1* helps to regulate, are critical for proper cell–cell junction functioning, receptor localization (e.g., Frizzled receptors), and the orientation of polarity proteins (e.g., Prickle). These processes are essential in maintaining the cytoskeletal arrangement, which in turn supports proper cellular organization and polarization during cardiac morphogenesis. Disruptions in microtubule dynamics and PCP signaling could lead to abnormal trabeculation and the failure of myocardial compaction, key features of LVNC [25,26,27].

During cardiac embryogenesis, the *MTUS1* gene affects the microtubule stability and other proteins involved in adequate cell polarization, an essential process for myocardial compaction, and could therefore be associated with congenital cardiomyopathies, including LVNC [5,22]. Furthermore, it was shown that the *MTUS1* genetic variant c.2617A>C (p. Asn873His) in HEK-293 cell lines reduces the extent of noncompaction through enhanced polarization and exerts a protective effect in the development of LVNC; since microtubule overstabilization is also observed in advanced heart failure, variations in *MTUS1* might have not yet described roles in cardiac functioning [3,5].

In addition to LVNC, our patient also had congenital cataracts, which are associated with numerous diseases and genetic abnormalities [28]. In our case, the cataract was bilateral, and it has been shown that 25% of congenital bilateral cataracts are associated with systemic disease [29]. However, none of the close relatives had any eye problems, and the genetic analysis of the whole genome did not reveal any pathological variants in genes associated with the development of cataracts. The role of the *MTUS1* variant in cataracts is not known, although, in mouse embryogenesis, multiple isoforms of *MTUS1* are expressed in different parts of the developing eye and in the vasculature around the eye, indicating a possible role of *MTUS1* in eye pathologies [30].

The pathological findings in the postmortem autopsy, including an abnormal mitochondrial morphology and muscle biopsy results, along with bilateral congenital cataracts, in the presented case report suggest the possibility of an underlying systemic disease as the cause of LVNC. However, despite extensive diagnostic efforts, including the comprehensive genetic analysis of both the nuclear and mitochondrial genomes, we were unable to fully explain the clinical presentation in our patient.

The association between the identified *MTUS1* variant and the underlying heart condition (ascending aortic aneurysm) in the patient’s father is unclear. However, since the pathophysiological mechanisms of aortic aneurysms and LVNC differ, the heart condition in the father may also be an incidental finding. Further research is needed in this area.

Importantly, the patient’s brother also carried the same two genetic variants in the *MTUS1* gene and remained in good health at the age of five. Since both variants have a substantial impact on the protein structure (an early stop codon and the deletion of an entire exon), our observations suggest that *MTUS1* variants alone do not cause LVNC or manifest in any major developmental disorder, and we suggest that additional factors, possibly genetic or environmental, may be required for the clinical manifestation of LVNC.

## 4. Conclusions

In conclusion, we analyzed two variants in the *MTUS1* gene that significantly affect the protein structure, but these variants alone may not be sufficient to cause LVNC or any other major developmental defect observable in childhood. Given the limited understanding derived from the literature and our own findings, the precise role of *MTUS1* in our patient’s unique phenotype remains largely unknown, and additional investigations are required to fully understand the potential clinical effects of *MTUS1* mutations later in life.

## Data Availability

Data are available upon request.
